# Colorimetric LPMO assay with direct implication for cellulolytic activity

**DOI:** 10.1186/s13068-021-01902-4

**Published:** 2021-02-27

**Authors:** Søren Brander, Stine Lausten, Johan Ø. Ipsen, Kristoffer B. Falkenberg, Andreas B. Bertelsen, Morten H. H. Nørholm, Lars H. Østergaard, Katja S. Johansen

**Affiliations:** 1 Department of Geosciences and Natural Resource Management, University of Copenhagen, 1958 Copenhagen, Denmark; 2 Department of Plant and Environmental Sciences, University of Copenhagen, 1871 Copenhagen, Denmark; 3 The Novo Nordisk Foundation Center for Biosustainability, Technical University of Denmark, 2800 Kongens Lyngby, Denmark; 4 Novozymes A/S, 2800 Kgs. Lyngby, Denmark

**Keywords:** Lytic polysaccharide monooxygenase, Enzyme assay, Phenolphthalein, Dehydroascorbate, High throughput, Cellulose

## Abstract

**Background:**

Lytic polysaccharide monooxygenases (LPMOs) are important industrial enzymes known for their catalytic degradation of recalcitrant polymers such as cellulose or chitin. Their activity can be measured by lengthy HPLC methods, while high-throughput methods are less specific. A fast and specific LPMO assay would simplify screening for new or engineered LPMOs and accelerate biochemical characterization.

**Results:**

A novel LPMO activity assay was developed based on the production of the dye phenolphthalein (PHP) from its reduced counterpart (rPHP). The colour response of rPHP oxidisation catalysed by the cellulose-specific LPMO from Thermoascus aurantiacus (TaAA9A), was found to increase tenfold by adding dehydroascorbate (DHA) as a co-substrate. The assay using a combination of rPHP and DHA was tested on 12 different metallo-enzymes, but only the LPMOs catalysed this reaction. The assay was optimized for characterization of TaAA9A and showed a sensitivity of 15 nM after 30 min incubation. It followed apparent Michaelis–Menten kinetics with *k*_cat_ = 0.09 s^−1^ and *K*_M_ = 244 µM, and the assay was used to confirm stoichiometric copper–enzyme binding and enzyme unfolding at a temperature of approximately 60 °C. DHA, glutathione and fructose were found to enhance LPMO oxidation of rPHP and in the optimized assay conditions these co-substrates also enabled cellulose degradation.

**Conclusions:**

This novel and specific LPMO assay can be carried out in a convenient microtiter plate format ready for high-throughput screening and enzyme characterization. DHA was the best co-substrate tested for oxidation of rPHP and this preference appears to be LPMO-specific. The identified co-substrates DHA and fructose are not normally considered as LPMO co-substrates but here they are shown to facilitate both oxidation of rPHP and degradation of cellulose. This is a rare example of a finding from a high-throughput assay that directly translate into enzyme activity on an insoluble substrate. The rPHP-based assay thus expands our understanding of LPMO catalysed reactions and has the potential to characterize LPMO activity in industrial settings, where usual co-substrates such as ascorbate and oxygen are depleted.

**Supplementary Information:**

The online version contains supplementary material available at 10.1186/s13068-021-01902-4.

## Background

Lytic polysaccharide monooxygenases (LPMOs) break down polysaccharides, but they are different from most lytic enzymes in the sense that they do not have a substrate-binding groove, tunnel or pocket [1, 2]. Instead, LPMOs have a flat, unrestricted binding site that allows bulky and insoluble substrates such as crystalline polysaccharides to bind to the active site [3]. The active site is defined by a copper ion that is bound by a N-terminal histidine, an internal histidine and the N-terminus in a conformation that is known as the copper histidine brace [4]. The catalytic mechanisms of LPMOs are still the subject of scientific debate. However, it is generally accepted that LPMO activity requires an electron-donating co-substrate (also called an electron donor or a reducing agent), an electron-accepting co-substrate (O_2_ or H_2_O_2_), and a copper co-factor to oxygenate and thus break the internal glycosidic bonds of polysaccharides [5]. Significantly, LPMOs work in synergy with polysaccharide hydrolases and thereby accelerate the decomposition of complex biomaterials such as lignocellulose. LPMOs have been shown to degrade isolated polysaccharides such as cellulose [6], chitin [7, 8], starch [9] and xylan [10] in the presence of electron-donating co-substrates such as ascorbate, gallic acid or the liquid fraction from hydrothermally pretreated lignocellulose [4]. The products are oxidised and un-oxidised poly- and oligosaccharides.

Product analysis of LPMO activity on an insoluble oligosaccharide substrate is time consuming and often requires several hands-on steps and lengthy HPLC protocols [11]. Mass spectroscopy and gel-based methods [4] have also provided evidence for LPMO catalysis of glycosidic bonds in recalcitrant polysaccharides. Such advanced methods can be left out if very detailed information about the product profile is not required. This is for instance the case when LPMOs are assayed in combination with cocktails of hydrolytic enzymes for full saccharification of lignocellulose [12]. Monosaccharide quantification by HPLC, traditional colorimetric reducing sugar assay [13, 14], or Brix analysis [15] may be sufficient in such studies. Production of oxidised polysaccharides is a hallmark of LPMO activity and methods to specifically quantify these products have been developed. In one method, the increased nickel affinity of oxidised sugars was used in a colorimetric assay [16] and, in another method, these sugars were hydrolysed to the monomeric form and quantified by the D-gluconic acid assay kit from Megazymes [17].

**Fig. 1:**
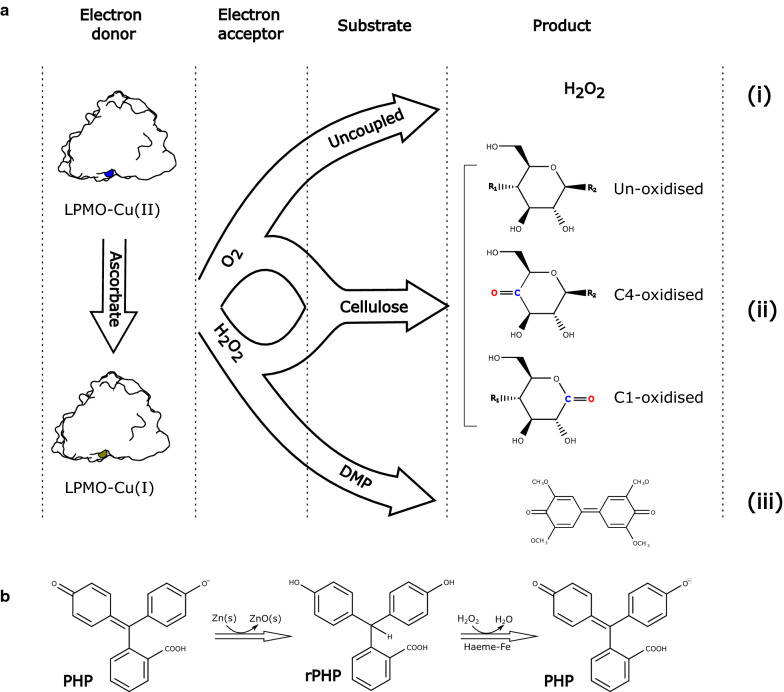
Schematic overview of reagents and products of relevance in the present work. **a** Illustration of the generally accepted reaction paths for cellulose-specific LPMOs. The reaction is preceded by the reduction of the active site copper by an electron donor, here exemplified by ascorbate. (i) When no substrate is present, the cuprous LPMO is reoxidised by the electron acceptor O_2_ and produces H_2_O_2_ which can be quantified, e.g., using the peroxidase-based Amplex® Red fluorescence assay. (ii) When cellulose substrate is present, O_2_ or H_2_O_2_ is used as the electron acceptor to oxygenate and ultimately cleave the cellulose to produce a mixture of un-, C1- or C4-oxidised sugars of various lengths. Soluble products can be separated by HPLC. (iii) In the presence of DMP or the related hydrocoerulignone, H_2_O_2_ is the preferred electron acceptor to oxidise the substrate to the colorimetric coerulignone, which can be measured by absorption at 469 nm. **b** Illustration of the interconversion between PHP and rPHP as utilised for the Kastle–Meyer presumptive blood test in forensic science. The Kastle–Meyer reagent is first prepared by reducing PHP with zinc dust, and it is then applied to, for instance a textile swab with 0.1 M H_2_O_2_ at pH 12. An immediate pink colour indicates the presence of blood

In the absence of a polysaccharide substrate, LPMOs produce H_2_O_2_ in an uncoupled reaction with suitable electron donors such as ascorbate [18–20]. H_2_O_2_ can be quantified in a peroxidase-coupled assay with Amplex® Red substrate [21]. Reaction progress data can be conveniently obtained directly by fluorescence spectrophotometry, e.g., in a microtiter plate format. However, the method is prone to false positives from free copper, which produces H_2_O_2_ at a higher turnover frequency (TOF) than LPMO-bound copper [22, 23]. In an alternative approach, assays to quantify the intrinsic peroxidase-like activity of LPMOs have been developed using 2,6-dimethoxyphenol (DMP) [24] or the oxidised form of it (hydrocoerulignone) [25] as substrate. These are very sensitive and high-throughput assays that can be used to compare LPMO containing samples of similar composition, e.g., during enzyme purification or for temperature stability tests. However, they require great care to prevent unwanted cross-linking and autoxidation reactions to take place. These DMP assays do not correlate with the enzymatic activity on polymeric substrates such as cellulose. Figure 1a illustrates known LPMO reactions.

Here we describe the development of a simple colorimetric assay that correlate with lytic activity of LPMOs on cellulose. We hypothesized that a good oligosaccharide mimic would have to be organic, hydrophilic, and contain a labile hydrogen. Triarylmethanes appeared to be good candidates due to their bulk, colour, and their central methyl group, where deprotonation is stabilized by the three aromatic rings. Reduced phenolphthalein (rPHP) is a colourless triarylmethane that is used in forensic science to indicate the presence of blood residues. Trace amounts of haemoglobin in a blood stain readily catalyse the oxidation of rPHP in 0.1 M H_2_O_2_ at pH 12 to give a strong pink signal within 10 s, in what is known as the Kastle–Meyer presumptive blood test [26] (see Fig. 1b). In this study, we show that LPMOs can oxidise rPHP at neutral pH through a mechanism that is enhanced by dehydroascorbate (DHA). The product is the well-known pH indicator phenolphthalein (PHP) which is quantified in a stable and sensitive enzyme assay.

## Results

### Initial screening for chromogenic LPMO substrates

Reduced and colourless counterparts of some triarylmethane dyes; PHP, fluorescein, phenol red, crystal violet and thymolphthalein, were readily obtained by boiling with zinc dust using a protocol similar to the preparation of the Kastle–Meyer reagent (Fig. S1). Of the tested dyes, rPHP showed the best stability against autoxidation, and was thus selected for optimization as an LPMO assay substrate. The initial colour screens showed slow enzyme-dependent oxidation of rPHP to PHP in the absence of ascorbate. The commercially available 2,7-Dichlorofluorescin diacetate (DCFH-DA) is an example of a triarylmethane, where enzyme-dependent oxidation is only slightly faster than autoxidation (Fig. S2).

**Fig. 2:**
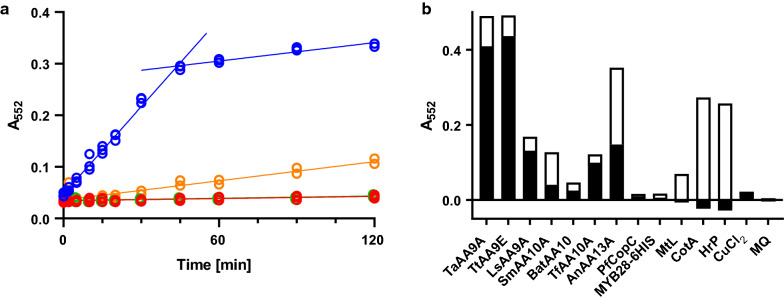
Effect of DHA on the formation of PHP as measured by absorption at 552 nm: **a** Progress of the rPHP assay was followed by stopping the reaction with 200 mM Na_2_CO_3_ pH 10.3 at various times (0–120 min) using the spectrophotometers build in injector module. rPHP was oxidised by 0.3 µM Cu-TaAA9 (orange), supplemented with 100 µM DHA (blue) or 100 µM ascorbate (green). rPHP oxidation by 0.3 µM CuCl_2_ supplemented with 100 µM DHA is shown for comparison (red). DHA appears to enhance the oxidation of rPHP. **b** Comparison of the DHA-enhancing effect on rPHP oxidation for 12 different proteins. LPMOs, were loaded with CuCl_2_ and 1 µM of each enzyme was incubated for 30 min at 40 °C with or without DHA. The enhancing effect of DHA is shown in black and the absorbance without DHA is shown in white bars

Figure 2a shows progress curves for the oxidation of rPHP to PHP in citrate phosphate buffer at pH 7.25 catalysed by 300 nM of the cellulose active LPMO from *Thermoascus aurantiacus* (TaAA9A). Under these conditions, the reaction proceeds with a reaction rate of 0.0029 s^−1^, which makes for a significant but very slow assay. In one experiment, 100 µM ascorbate was included to investigate the potential effect of this known LPMO electron-donating compound. Surprisingly, the effect of ascorbate was complete inhibition of the rPHP oxidising reaction. The disappearance of ascorbate was followed by measuring the specific absorption band at 265 nm (Fig. S3) and this result is discussed further below. In parallel with the ascorbate experiments, the effect of 100 µM DHA on rPHP oxidation by 300 nM TaAA9A was tested. The result was an increase in the production of PHP by a factor of ten to a reaction rate of 0.030 s^−1^. The enhancing effect stops after 45 min and is followed by a slow phase similar to the reaction without DHA. The DHA dependent activity spurred us to further develop rPHP oxidation as a convenient spectroscopic LPMO assay.

### The specificity of the rPHP enzyme assay

The enhanced rPHP oxidising activity of TaAA9A with DHA was surprising and raised the question of whether other enzymes react in a similar way. Figure 2b shows the rPHP oxidising activity of seven LPMOs, a copper chaperone as one negative control, and a his-tagged protein as a second negative control. Two laccases and horseradish peroxidase were also tested as these enzyme classes generally oxidise phenolic compounds, as exemplified by their positive response in the DMP assay [24], and they are also likely candidates to oxidise rPHP. (See the Materials and methods section for details of names and sequence identifiers.) All LPMOs tested oxidise rPHP in a DHA dependent reaction. However, the DHA-enhancing effect on the two chitinolytic LPMOs SmAA10 and BatAA10 was small. The starch specific LPMO AnAA13 stands out with a relatively large rPHP oxidation without DHA. The copper chaperone PfCopC binds copper in a histidine brace similar to LPMOs, but has been shown not to oxidise ascorbate under similar conditions [23]; neither does it oxidise rPHP. His-tags for protein purification bind copper with K_D_ < 1 nM [27] and might give rise to false positives in a redox assay. MYB28 is a non-enzymatic protein that was expressed and purified with a his-tag and, as expected, it did not oxidise rPHP. The laccases (CotA) and (MtL) as well as horseradish peroxidase (HrP) did oxidise rPHP; however, the rPHP oxidising activity of these enzymes was not enhanced by DHA. Under the conditions investigated, the DHA-dependent oxidation of rPHP to PHP appears to be LPMO specific.

### Effect of pH and buffer on rPHP oxidising activity of TaAA9A

Several buffer compositions; ethylmorpholine, MOPS, citrate, phosphate, citrate–phosphate and citrate–phosphate-borate were tested to investigate potential buffer effects and to determine the optimum pH for the assay (Fig. S4a, b). The response of the assay increased monotonically with pH and we decided to continue development of the LPMO assay using citrate–phosphate buffer at pH 7.25. This condition showed good assay response and stability. The effect of ionic strength was tested by addition of NaCl in range of concentration 0–2 M. The assay response is unchanged in the 0–0.1 M range after which it starts to decline (Fig. S4c). Similarly, the assay response is stable to changes in the buffer concentration (Fig. S4d).

### Dose response of assay components

**Fig. 3:**
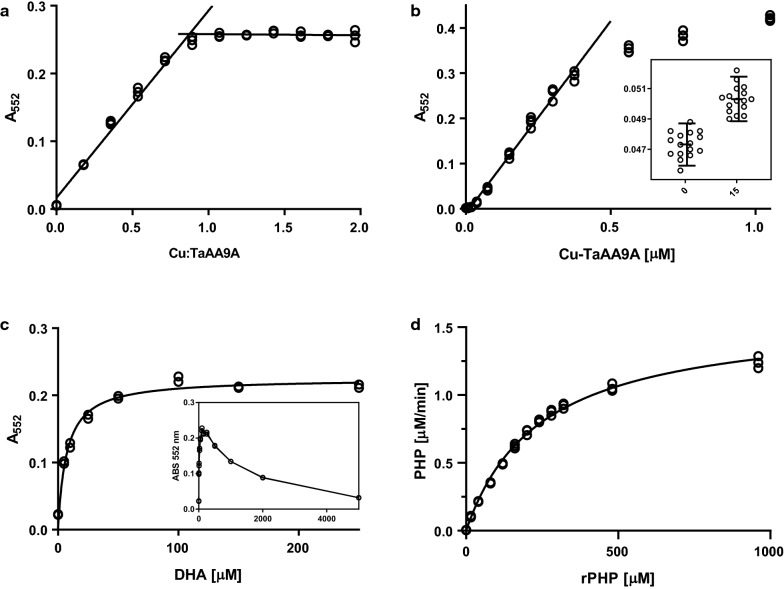
Dose–response curves for different components of the rPHP-based LPMO assay determined by the absorbance at 552 nm after 30 min of incubation at 40 °C. Reaction conditions are 300 nM TaAA9A, 200 µM rPHP, 100 µM DHA, and 25 mM citrate–phosphate buffer pH 7.25 with the following modifications: **a** The relative copper loading of the TaAA9A was varied (0–600 nM CuCl_2_). **b** The concentration of Cu-TaAA9A was varied (0–1 µM) and was found to be linear in the lower range 0–300 nM. Measurements on 16 samples at 0 or 15 nM enzyme concentrations are shown in the insert. The horizontal lines mark the σ = 1.645 thresholds. **c** The DHA concentration was varied (0–5.0 mM) and a plateau is observed in the range 50–250 µM. **d** The rPHP concentration was varied (0–1.0 mM). The formation rate of PHP was calculated and plotted together with the best fitting Michaelis–Menten kinetics curve, described by *K*_M_ = 244 µM, *V*_max_ = 1.6 µM/min, *k*_cat_ = 0.09 s^−1^ with an *R*^2^ of 0.99

First, the concentration of TaAA9A was determined by quantitative amino acid. This method is time-consuming but reputable and precise. In this case, the enzyme concentration calculated from tryptophan absorption values at 280 nm was 30% higher than this quantitative HPLC method. The assay activities of Cu-TaAA9A samples at various ratios between copper and enzyme were tested and showed a titration point at stoichiometric loading (Fig. 3a). This observation confirms that the catalysed reaction is dependent on the copper at the copper-histidine brace and it also indicates a stable assay that is not significantly affected by free copper. Ethylenediaminetetraacetic acid (EDTA) and bicinchoninic acid (BCA) have very high binding affinities for copper, but the addition of 100 µM of either chelator did not interfere with the assay at all (Fig. S5a). This is in agreement with the previously reported copper binding affinity of TaAA9A with k_D_ < 1 pM [4].

The assay response was linear with Cu-TaAA9A concentration up to 300 nM. At higher concentrations of enzyme another assay component becomes limiting (Fig. 3b). The insert in Fig. 3b shows the raw assay readings from 16 independently prepared samples at either 0 nM or 15 nM together with σ = 1.645 demarcations that correspond to 90% of their populations in a normal distributed sample set. The two populations do not overlap and thus set the sensitivity of the assay to 15 nM Cu-TaAA9A with 5% of samples expected to be false negatives. Breslmayr et al. also used this criterion to define the lower limit of detection in the DMP assay [24, 25]. In combination, the two limits show that the rPHP assay is sensitive and linear in the range of 15–300 nM TaAA9A. In an attempt to prolong the fast phase of the rPHP oxidation, we turned to investigate dose–response of DHA on rPHP oxidation. Oxidation of rPHP, by 300 nM TaAA9A in 30 min, at various DHA concentrations is shown in Fig. 3c. The assay response increases with DHA concentration until it reaches a plateau between 50 and 250 µM after which the response starts to decrease. The non-monotonic dependency on DHA complicates kinetic analysis as it is uncertain whether DHA is saturated in the assay; however, a traditional Michaelis–Menten kinetic analysis was attempted by varying the concentration of rPHP at a constant concentration of 100 µM DHA. The concentration of rPHP was determined from a standard curve of PHP solutions in reaction conditions, and the reaction rates interpolated from single point measurements (Fig. S6). The assay output fits well to the model with K_M_ = 244 µM and k_cat_ = 0.09 s^−1^ as shown in Fig. 3d.

### The temperature profile of rPHP oxidation can be used to identify new LPMO co-substrates

**Fig. 4:**
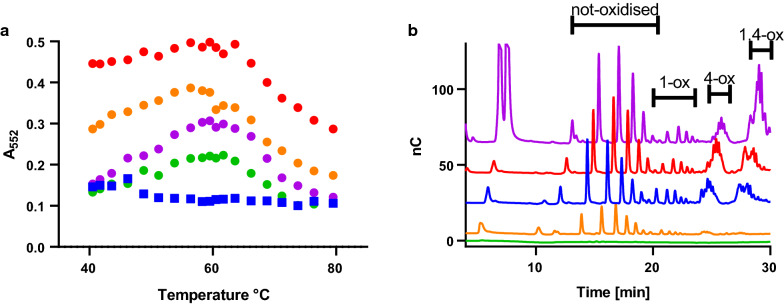
Activity profiles of TaAA9A with various co-substrates. **a** The rPHP oxidation temperature profiles using potential boosting compounds, 100 µM GSH (orange), 100 µM DHA (red), 1.25 mM fructose (purple), Milli-Q water (green), and ascorbate (blue). **b** Cellulose degradation products analysed by HPAEC after incubation of 0.75 µM TaAA9A with 0.4% PASC in 25 mM citrate phosphate buffer at pH 7.25 and 50 °C. The co-substrates were added in 1 mM concentration and the samples were incubated for 1 h, except for the sample with fructose, which was incubated for 23 h. Chromatograms are representative of duplicate experiments and colour coded as in (**a**). Glucose and fructose monomers have retention times of about 5 min. Peaks at 12–20 min are from not-oxidised sugars, peaks at 20–25 min are from C1-oxidised sugars, while peaks at 28 min are from two-times oxidised sugar products

Having established that the rPHP assay is a sensitive and specific LPMO assay, we wanted to investigate if it is also indicative of activities relevant to the industrial application of these enzymes. Figure 4a shows the results of a slightly modified assay in which the samples were incubated in a thermocycler with a gradient of temperatures in the range 40–80 °C for just 10 min. The temperature profile of the rPHP reaction showed an optimum at 60 °C, which is close to the reported melting temperature of TaAA9A of 62°C [22]. This assay is clearly dependent on the thermal stability of the enzyme. Using this version of the assay, we tested a few other compounds with potential to enhance oxidation of rPHP by TaAA9A instead of DHA. Ascorbate quenched the assay at all temperatures, while the milder reductant glutathione (GSH) enhanced rPHP oxidation at all temperatures. Glucose did not affect the assay response (data not shown). However, fructose, another monosaccharide, did enhance the oxidation of rPHP by TaAA9A at temperatures higher than 40 °C. All monosaccharides are reducing in, e.g., Fehling copper solution in highly alkaline conditions, but at circumneutral pH fructose is the better reductant [28]. In this aspect, ascorbate, GSH and fructose exhibit different behaviour as reductants and the temperature profiles provides a visual tool to inspect these differences that are not apparent from just measurements at 40 °C. The relatively wide temperature profile can be explained by the reported ability of TaAA9A to refold after heat treatment [22].

### Degradation of cellulose by TaAA9A in the rPHP assay conditions

The enhanced rPHP oxidation with DHA and inhibition hereof with ascorbate is in contrast to the expected effects in a cellulose degradation experiment. In the light of this discrepancy, we moved to investigate how the rPHP assay conditions affected degradation of phosphoric acid swollen cellulose (PASC). Figure 4b shows the high-performance anion-exchange chromatography (HPAEC) chromatograms of saccharide products after degradation of 0.4% PASC by 0.75 µM TaAA9A incubated at pH 7.25 with GSH, DHA, or ascorbate for 1 h and fructose for 23 h. Ascorbate and GSH enabled the degradation of cellulose as shown by a pattern of both unoxidised and oxidised oligomeric products. Fructose and DHA were also able to facilitate cleavage of cellulose by TaAA9A, albeit at a relatively low rate. Another series of experiments showed that DHA enable cellulose cleavage at pH > 5 and fructose at pH > 7 (Fig. S7).

### The role of oxygen and H_2_O_2_ in DHA-enhanced oxidation by TaAA9A

**Fig. 5:**
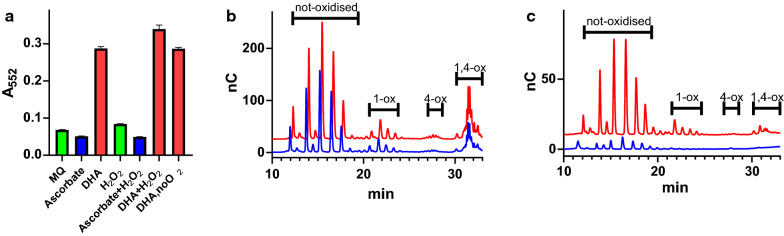
Activity of TaAA9A with ascorbate, DHA, O_2_ and H_2_O_2_. **a** rPHP oxidation assay with various co-substrates: Milli-Q water (black), 100 µM ascorbate (blue), 100 µM DHA (red) in combinations with or without 100 µM H_2_O_2_. The sample with “no O_2_” was prepared, incubated and stopped inside a strictly anaerobic glovebox. **b**, **c** Cellulose degradation analysed by HPAEC after 23 h incubation using ascorbate (blue) or DHA (red) as co-substrates. Samples were as described in Fig. 4, but prepared in double volumes and inside a strictly anaerobic glovebox. Samples were then split in two, one portion was removed from the glovebox and incubated in normal atmosphere **b**, the other portion incubated in the anaerobic environment **c**

The effect of varying the concentration of O_2_ or H_2_O_2_ on rPHP oxidation was also tested. For this experiment, the TaAA9A catalysed oxidation of rPHP was studied in a combination of conditions with and without ascorbate, DHA, O_2_, and H_2_O_2_ (Fig. 5a). A small increase in the DHA-dependent rPHP oxidation was seen when adding H_2_O_2_, while ascorbate stop the oxidation both with and without H_2_O_2_. Surprisingly, the rPHP oxidation with DHA was equally efficient when carried out in an anaerobic glovebox or in normal atmosphere. This is in contrast to the O_2_ limited oxidation of ascorbate by TaAA9A as demonstrated by a linear relationship between O_2_ concentrations at rate of ascorbate oxidation (Fig. S8). The effect of these conditions on TaAA9A degradation of PASC was then tested. Samples with 0.4% PASC, 0.75 µM TaAA9A, and 1 mM ascorbate or 1 mM DHA, were prepared in strict anaerobic conditions. Samples were split in two, one part was taken out of the glovebox and incubated in normal atmosphere, and the other incubated anaerobically at 50 °C for 23 h. Figure 5b shows the HPAEC data for the PASC degradation in normal atmosphere , and samples with ascorbate or DHA as co-substrates produce similar degradation profiles. A clear set of peaks corresponding to not-oxidised products are seen. Also, the two-times oxidised sugars are prominent products, while C4 oxidised products are hardly detectable. The HPAEC chromatograms for the anaerobic condition (Fig. 5c), show only small amounts of products with ascorbate as co-substrate. However, the sample with DHA as co-substrate produced roughly 50% not-oxidised products compared to the aerobic condition but no C4-oxidised and two-times oxidised products.

### The role of ascorbate and DHA in rPHP oxidation by TaAA9A

The distinct absorption at 265 nm by ascorbate can be used to quantify the concentration of ascorbate in solution. The absorbance at this wavelength was followed during the incubation of rPHP reaction mixtures until the addition of the stop buffer to the samples described in Fig. 2a. Under these conditions, it took about 10 min for the 100 µM ascorbate to be completely oxidised (Fig. S3a). This is probably the result of the uncoupled reaction of TaAA9A with ascorbate and O_2_ to produce H_2_O_2_ and DHA [23]. Thus, a general antioxidant effect of ascorbate appears to be inconsistent with the complete inhibition of rPHP oxidation for at least 120 min by ascorbate. Both the slow and fast phase of the reaction were inhibited and the most plausible explanation is that TaAA9A was modified. Indeed, ascorbate appeared to result in degradation of TaAA9A as shown by SDS PAGE (Fig. S3b).

## Discussion

### The rPHP assay is a fast and sensitive method

The product of this assay is the well-known pH indicator PHP, and the enzyme activity can be quantified after only 30 min of incubation by measuring the absorption at 552 nm. The assay is highly sensitive and reliably detects levels of TaAA9A down to 15 nM. The rPHP substrate used is very stable in solution and can tolerate heat and extreme pH, which is a crucial factor in the making of a robust assay. Another important factor is that presence of free copper does not affect the assay output. This allows determination of, not only the LPMO activity and stability, but also the relative enzyme copper loading and the concentration of active LPMO.

### Kinetic parameters for rPHP oxidation by TaAA9A

We have shown that rPHP is a substrate for LPMOs from families AA9, AA10, and AA13. The kinetic constants were determined for TaAA9A and the calculated k_cat_ of 0.09 s^−1^ is similar to the TOF of ascorbate oxidation (0.14 s^−1^) reported for this enzyme [23]. It is likewise very similar to the k_cat_ for the cleavage of glucosidic bonds by another fungal LPMO, namely LsAA9A [1]. The maximum oxidation rate of rPHP by TaAA9A thus progresses at a rate similar to the oxidation of other known substrates. The apparent K_M_ of 244 µM for the rPHP substrate exceeds the fixed concentration of the co-substrate DHA, and may, therefore, not reflect the actual binding affinity. A similar problem was encountered in the peroxidase-like assays described by Breslmayr et al. [24, 25], where they were unable to saturate the reaction with H_2_O_2_. At 100 µM H_2_O_2_ they reported K_M_ in the range 3.6–100 mM for hydrocoerulignone or DMP substrates. Comparison of apparent K_M_ values suggests that rPHP is a better-matching substrate. Values of k_cat_ around 0.1 s^−1^ are much lower than the 10 s^−1^ reported for cleavage of glucosidic bonds when using H_2_O_2_ as that co-substrate [29]. The addition of H_2_O_2_ to the rPHP assay mixture under ambient conditions increased the amount of PHP produced in 30 min, but only by about 20%. Anoxic conditions do not seem to have any effect on the oxidation of rPHP by TaAA9A. In fact, neither O_2_ nor H_2_O_2_ appear to be essential for the reaction when DHA is used as the co-substrate.

### rPHP assay informs on the cellulolytic activity of LPMOs

Surprisingly, the rPHP assay does not work with the commonly used electron-donating co-substrate ascorbate, which is otherwise known to reliably promote LPMO degradation of cellulose. This can be explained by instability of TaAA9A towards ascorbate under the given conditions. However, the oxidation of rPHP is instead enhanced by DHA, which is a product of ascorbate oxidation. DHA will thus be readily available in cellulose assays in which ascorbate is co-substrate. In the light of the new-found reactivity with LPMO, we investigated whether DHA could also promote cellulose degradation. It was found that DHA is a reasonable co-substrate for cellulose cleavage by TaAA9A acting on both cellulose and rPHP substrate with similar pH profiles. In addition, DHA was able to promote cellulose degradation in anaerobic conditions, while ascorbate was unable. This could have a profound impact on how cellulose assays are interpreted in the future. It appears that the DHA-dependent rPHP activity is more than simply a side activity, but actually infers understanding of how the enzyme behaves on the complex cellulose substrate. Interestingly, the anaerobic cellulose degradation with DHA produced a HPAEC profile with hardly any peaks from oxidised sugars.

### LPMO specific assay

A total of 12 different enzymes were tested in the rPHP assay, and only the seven LPMOs showed increased activity when co-incubated with DHA. The rPHP assay, therefore, has the potential to be an LPMO-specific activity test. A distinction was seen between AA9, AA10, and AA13 LPMOs, where the cellulose specific fungal AA9 LPMOs showed the highest activity. This may reflect an intrinsic higher activity of these enzymes, but can also be the result of the assay being optimized for TaAA9A. The two LPMOs with the least assay response was SmAA10 and BatAA10, two chitin active bacterial LPMOs. This difference in assay response may reflect a better binding of rPHP to cellulose or starch active LPMOs. Another structural difference that can explain the smaller DHA-effect by SmAA10 and BatAA10 is found at the copper binding site. Copper in the chitinolytic LPMOs has a phenylalanine ligand in the distal position, while the rest of the tested LPMOs have a tyrosine. It is possible that the tyrosine sidechain enables an internal electron pathway to a co-substrate site, where, e.g., DHA can bind.

To further validate the rPHP assay as an alternative to cellulose assay, we investigated the surprising observation that fructose also enhanced the rPHP oxidising activity of TaAA9A, albeit at a concentration ten times higher than DHA. The use of fructose as co-substrate in the cellulose assay resulted in oligosaccharide under similar conditions given by the rPHP assay. The two assays both showed that fructose-driven LPMO activity required a pH higher than 7 to provide a significant product yield, while the effect of DHA started at pH higher than 5. The fact that we could use the rPHP assay to identify new activity conditions for TaAA9A, and then successfully use the same parameters in a cellulose degradation assay, shows that the rPHP assay has the potential to expand our understanding of the primary activity of LPMOs and not only their side activities.

### rPHP is a cellulose mimic

**Fig. 6:**
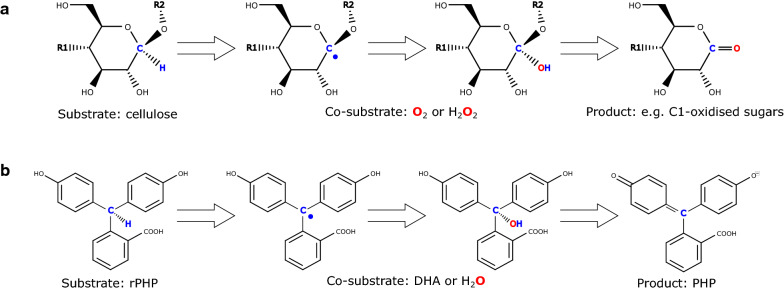
Proposed chemical principles for the LPMO oxidative activity on cellulose and rPHP. **a** Various mechanisms have been proposed for the LPMO-catalysed degradation of cellulose but a hydrogen abstraction event from the C1 or C4 carbon of a saccharide entity is central to the reactions. In this reaction scheme, a C1 carbon is highlighted in blue. Hydrogen abstraction leaves a carbon radical that readily reacts with an electron acceptor such as O_2_ or H_2_O_2_ to form C1-oxidised and non-oxidised products. **b** A similar mechanism for rPHP oxidation is reasonable. Abstraction of hydrogen from the central methyl group leaves behind a carbon radical which is hydrolysed to produce a carbinol. The carbinol is known from over-titration of PHP, and the neutral form of PHP is adapted in the neutral pH buffer of the rPHP assay

rPHP was initially chosen for investigation as a potential LPMO substrate, because it can mimic the chemical principles of LPMO cleavage of glucosidic bonds. Specifically, rPHP is a large flat molecule with a central weak C-H bond from which an LPMO might abstract the hydrogen in what resembles the initial attack on cellulose. The observed similarity between rPHP and cellulose as LPMO substrates indicates that such a mimicry is reasonable. Figure 6 outlines a hypothetical reaction path for LPMO oxidation of rPHP together with a simplified reaction path for cellulose degradation that show how the two reactions can be explained by similar mechanisms. DHA is a somewhat unstable compound and it is possible that one of the degradation products is the actual compound that reacts with LPMO [30]. Further investigations are needed to characterize the role of DHA, but in principle it can provide both electron donors and acceptors. The rPHP assay represents new enzymatic activity without prior description, but in preparation of this manuscript, we discovered the work by Kalinich et al. who reports how gamma rays induce oxidation of the E-vitamin mimic Trolox that subsequently goes on to abstract a hydrogen from 2,7-dichlorofluorescin diacetate (DCFH-DA) and ultimately oxidise it [31]. Their system resembles the rPHP assay with DCFH-DA being a reduced triaryl methane similar to rPHP and the oxidised Trolox being a counterpart to DHA. Indeed, we found that DCFH-DA is also a substrate of TaAA9A in the presence of DHA (Fig. S2) Their work supports the rPHP assay hydrogen abstraction mechanism but it clearly warrants further investigation.

### The DHA-dependent LPMO activity is industrially relevant

The finding that LPMOs have different activities on ascorbate and DHA has more applied implications. When loading an LPMO with copper in the laboratory, it is easy to (accidentally or intentionally) overload the enzyme resulting in extra solvated copper. This is problematic in otherwise well-defined assays that use ascorbate as co-substrate. This is because ascorbate will readily react with free copper and O_2_ to produce DHA, H_2_O_2_, and several derived compounds [32]. This makes for a highly unpredictable composition of reagents in the assay. The DHA-enhanced oxidation of rPHP does not suffer from such complications.

Industrially relevant enzymatic saccharification studies indicate that LPMOs improve the monosaccharide yield even after days of incubation [12, 33]. These studies also showed that the content of dissolved O_2_ in the reaction mixture must be tightly controlled. The DHA-dependent LPMO activity points to a relatively slow but sustained LPMO reactivity that is not limited by the content of dissolved O_2_ or the activated oxygen species H_2_O_2_. (H_2_O_2_ has attracted much attention as an alternative LPMO co-substrate, but its expected lifetime in a complex reaction mixture is short, and its applicability as a co-substrate in a biomass reactor is debated [34, 35]). It is possible that other oxidised compounds than DHA have a similar effect on LPMO activity and thus enable prolonged LPMO activity. In a biomass reactor the co-substrates that enable LPMOs are derived from lignin and the existence of oxidised lignin compounds that activate LPMOs have been hinted at in the literature. For example, laccases and phenol oxidases can oxidise lignin monomers and enhance the LPMO catalysed degradation of cellulose in reactions taking place over 1–3 days [36, 37]. Another line of studies suggests that small lignin compounds enable LPMO activity by mediating electron transfer from bulk lignin [38]. However, the redox potentials of different lignin fractions and LPMOs were later measured [39] and the formal values imply that the suggested reversible electron transfer mechanism would run in reverse and produce oxidised lignin compounds without reducing the LPMO. It may well be, that the relatively slow LPMO activity, show-cased by rPHP oxidation, is a good proxy for LPMO reactions relevant to industrial settings.

## Conclusion

The LPMO assay described in this paper is reliable and high-throughput ready. The assay is based on the oxidation of rPHP to form the pink PHP dye, and the activity can be measured after 30 min of incubation. The assay is sensitive to 15 nM LPMO but insensitive to free copper, making it a useful tool for comparing LPMO samples and determining copper loading profiles and in situ temperature profiles.

The assay uses DHA as a co-substrate to specifically enhance oxidation of rPHP by LPMOs. This DHA-dependent effect was observed for cellulose and starch degrading LPMOs from families AA9, AA10 and AA13, while the two chitinolytic AA10s tested showed limited DHA-enhancement of PHP production.

The assay was used to identify DHA and fructose as co-substrates for LPMO activity, and the identified conditions were transferable to also induce activity with cellulose as the substrate. In this aspect, the rPHP assay is a rare example of a secondary activity assay that can provide information on the primary activity.

This rPHP-based assay also suggests that LPMOs can function as catalysts even at very low concentrations of O_2_ or H_2_O_2_ co-substrates. This is interesting as LPMOs are used commercially in reactions that proceed over several days, where such reactants may rapidly become depleted. The LPMO assay thus has the potential to characterise this slower LPMO activity, which is relevant in industrial settings.

## Methods

### Materials and enzymes

All chemicals were of the highest purity grade available and purchased from Sigma-Aldrich (Saint Louis, USA), unless otherwise stated. Glacial acetic acid, hydrogen peroxide and fructose were purchased from Merck (Darmstadt, Germany).

LPMOs from *Thermoascus aurantiacus* (TaAA9A), UniProtKB**:** G3XAP7, *Thermothielavioides terrestris* (TtAA9), UniProtKB: D0VWZ9, *Lentinus similis* (LsAA9A), UniProtKB: A0A0S2GKZ1, *Aspergillus nidulans* (AnAA13), UniProtKB: Q5B1W7, as well as the laccase from *Myceliophthora thermophila* (MtL), UniProtKB: G2QFD0 were kind gifts from Novozymes A/S (Bagsværd, Denmark). LPMOs from *Bacillus atrophaeus* (BatAA10), UniProtKB: A0A0H3E2X6, *Serratia marcescens* (SmAA10), UniProtKB: O83009, *Thermobifida fusca* (TfAA10A), UniprotKB: Q47QG3 were expressed using the “LyGo” platform as previously described [40]. Copper chaperone from *Pseudomonas fluorescens* (PfCopC), UniProtKB: A0A0D0TME7 [23] and laccase from *Bacillus subtilis* (CotA), UniProtKB: H8WGE7 [41] were expressed heterologously in *Escherichia coli* as previously described*.* All enzymes were purified to homogeneity before use. Horseradish peroxidase (HrP) was purchased from Sigma-Aldrich and used without further purification. LPMO concentrations were quantified after acid hydrolysis and separation of individual amino acids by ion-exchange chromatography [42].

A stock solution of rPHP was created by reducing PHP with zinc dust. For this, 1 g PHP, 4 g zinc dust, and 2.6 g NaOH were suspended in 200 mL water and boiled for 20 h with reflux. The rPHP solution was neutralized with 4 mL glacial acetic acid and boiled for another 2 h. The solution was brought to room temperature and zinc oxide was removed by decanting the rPHP solution into a new flask. Some fresh zinc dust was added to the rPHP and it was stored at 4 °C for over a year without loss of activity. The rPHP concentration was determined to be 16 mM by first oxidising a 1.25% solution to completion by HrP and comparing absorption at 552 nm to a standard curve for PHP (Fig. S6).

rPHP is commercially available under the trivial name phenolphthalin and we purchased a vial from Glentham Life Sciences (Corsham, UK). Activity of this commercial rPHP was similar to what we observed for the in-house prepared compound, although the assay response was lower.

### LPMO activity assay protocol

The rPHP assay went through several stages of optimization. However, during the preparation of this manuscript, the experiments were repeated using the optimized assay conditions: triplicates of 200 µL of 0.3 µM Cu-TaAA9A, 200 µM rPHP, 25 mM citrate–phosphate buffer at pH 7.25 and 100 µM DHA were incubated in a microtiter plate well for 30 min at 40 °C with shaking at 450 rpm. The assay colour was developed by adding 50 µL 1 M Na_2_CO_3_ at pH 10.3, and the absorption measured at a wavelength of 552 nm on a BioTek Synergy H1 plate reader. The components were combined in parts of 50 µL from fourfold concentrated stocks. Prior to the experiments the LPMOs stocks were incubated over night at 4 °C with stoichiometric amounts of CuCl_2_. This optimized assay condition was used as the core setup on which further characterisation of the assay was carried out by altering one or two parameters as described in the text. Some of the conditions needs further elaboration.

### Progress curve

The rPHP assay is discontinuous and the progress curves were measured by starting a series of technical replicate reactions and stopping them at different timepoints. These samples were not incubated in a thermomixer but rather inside the plate reader (preheated to 40 °C). At pre-programmed timepoints, the plate reader measured the ascorbate absorption at 265 nm before injection of 50 µL 1 M Na_2_CO_3_ to specified wells, shook the plate for 4 s and read the PHP absorption at 552 nm.

### Lower limit of detection

The lower limit of assay sensitivity was determined by analysis of 16 independently prepared samples. In practice, four assay plates were set up over the course of 2 days, each plate with four samples containing 0, 10 or 15 nM TaAA9A. Each sample was copper loaded individually and all pipetting carried out using a single channel pipette. Fresh stock of DHA was used for each plate. Standard deviations at *σ* = 1.645 were calculated using the STDEV function in Microsoft Excel 2016.

### Michaelis–Menten kinetics

rPHP concentrations were varied and the resulting assay measurement used in kinetic analysis using the Michaelis–Menten model: *v* = *E*_0_ × *k*_cat_ [*r*PHP]/(*K*_M_ + [*r*PHP]), where v is the rate of PHP formation and E_0_ the enzyme concentration. rPHP oxidation was assumed to be linear with time for all concentrations of rPHP and the conversion factor *v* = A_552_/(1.6 × 10^–2^ µM^−1^ × 30 min) was used (Fig. S6).

### Temperature optimum and enzyme stability

For the determination of the temperature profile of TaAA9A in the assay, the assay was scaled down to 100 µL and mixed in a PCR tube. The reactions were incubated in a thermocycler with temperature gradient capability in three runs: 40–60 °C, 50–70 °C, and 60–80 °C. After 10 min incubation, the tubes were put on ice and the reaction stopped with 25 µL 1 M Na_2_CO_3_. The samples were withdrawn from the PCR tubes and transferred to a low-volume microtiter plate for absorption measurements. Data showed that there was a significant edge effect from the thermocycler gradient and the outer data points were omitted giving a total of 30 data points in each series.

### Cellulose degradation

Duplicate mixtures of 0.75 µM Cu-TaAA9A, 0.4% PASC, 25 mM citrate phosphate buffer at pH 7.25 and various co-substrates (total 200 µL) were incubated at 50 °C with shaking at 850 rpm. The co-substrates ascorbate, DHA, glutathione, and fructose were added at concentrations of 1 mM, and the reaction mixtures were incubated for 1 h or 23 h. After incubation, the reactions were stopped by the addition of 50 µL 0.5 M NaOH and then passed through a 0.45 µm filter. The released saccharides were analysed by HPAEC on an ICS 5000 equipped with a PAD detector (Dionex, Sunnyvale, CA, USA) and a CarboPac PA1 column. Chromatography was carried out following the method of Westereng et al. [11]. Briefly, the saccharides were eluted in 0.1 M NaOH with a non-linearly increasing amount NaOAc.

### Anaerobic experiments

rPHP and cellulose assays were investigated in anaerobic environment. In preparation of the experiments, buffer, substrates, enzyme and stop solutions were placed with mild agitation in a rigid acrylic glovebox (Belle Technology UK Ltd) and the box was purged with N_2_ gas for at least 1 day. On the day of experiment, DHA and ascorbic acid were dissolved in the already purged buffer. During the experiments, the build-in oxygen meter showed values of 20–40 ppm O_2_.

### Detailed rPHP assay protocol

Step 1: Prepare an assay buffer: Dissolve 6 g Na_2_HPO_4_-7⋅H_2_O in 200 mL milliQ water and add citric acid to pH 7.25. Prepare a 800 µM rPHP solution. Prepare a 40 mM DHA solution (43.5 mg in 5 mL milliQ water) and dilute to 400 µM. Remaining DHA can be aliquoted and stored frozen. Prepare a stop solution: Dissolve 5.25 g Na_2_CO_3_ and 4.2 g NaHCO_3_ in 100 mL milliQ water and check that the pH is 10.3.

Step 2: Prepare enzyme solution: Equal volumes of 2.4 µM apo-enzyme and 2.4 µM CuCl_2_ are mixed and incubated overnight at 4 °C.

Step 3: Prepare an assay mix by combining equal volumes of assay buffer, rPHP solution, and DHA solution. In a microtiter plate, add 50 µL of LPMO sample to each well in a microtiter plate, and initiate the assay with 150 µL of the assay mix. Incubate the plate at 40 °C for 30 min while shaking at 450 rpm.

Step 4: Add 50 µL of stop buffer to each well and a pink colour will appear. Measure the absorption at 552 nm shortly after.

## Supplementary Information

**Additional file 1**: Supplementary information for colorimetric LPMO assay.

## Data Availability

All data are presented.
