# Supplementary material for: Colorimetric LPMO assay with direct implication for cellulolytic activity
Source: Biotechnol Biofuels. 2021 Feb 27;14:51. doi: 10.1186/s13068-021-01902-4 (PMC7916272; doi:10.1186/s13068-021-01902-4)
Supplement: Supplementary file 1 — Additional file 1: Supplementary information for colorimetric LPMO assay. [file 13068_2021_1902_MOESM1_ESM.docx]

# **Supplementary information**

Colorimetric LPMO assay with direct implication for cellulolytic activity

Søren Brander*^1^*, Stine Lausten*^2^*, Johan Ø. Ipsen*^2^*, Kristoffer B. Falkenberg^3^ , Andreas B. Bertelsen^3^ , Morten H. H. Nørholm^3^ ,Lars Østergård^4^, Katja S. Johansen*^1*^*

^1^Department of Geosciences and Natural Resource Management, University of Copenhagen, DK-1958 Copenhagen, Denmark

^2^Department of Plant and Environmental Sciences, University of Copenhagen, DK-1871 Copenhagen, Denmark

^3^The Novo Nordisk Foundation Center for Biosustainability, Technical University of Denmark, 2800 Kongens Lyngby, Denmark

^4^Novozymes A/S, Kgs. Lyngby, DK-2800, Denmark

*Corresponding author. Email: [ksj@ign.ku.dk](mailto:ksj@ign.ku.dk)


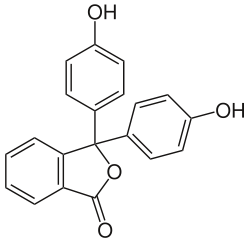

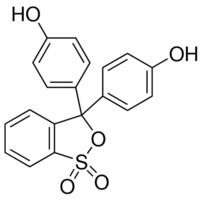

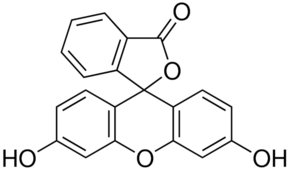

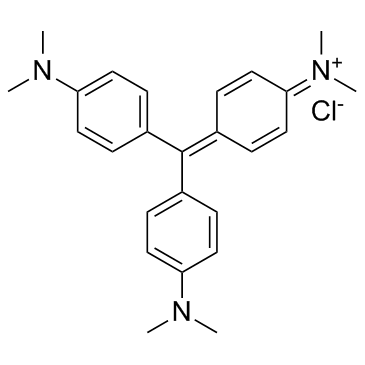

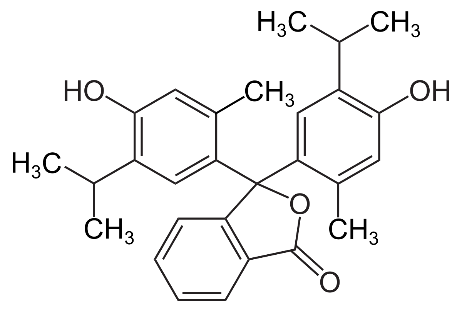

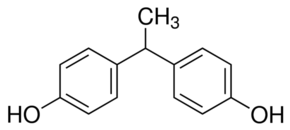


Phenolphthalein

Phenol red

Fluorescein

Thymolphthalein

Crystal violet

Bisphenol E

*Fig. S1: Aryl methanes that were reduced and tested as potential LPMO substrates. All structures are shown in the closed form, corresponding to their state in neutral pH. The putative substrates were reduced similar to what is described in the main text: 1 g aryl methane was boiled with 4 g zinc dust and 1.5 g NaOH. After 20 h the reactions were stopped by addition of 4 mL glacial acetic acid and the zinc was allowed to settle. These preparations were used in 1% concentrations in crude activity test with 10 µM TaAA9A, citrate-phosphate buffer pH 7.0 for 24 h with or without addition of 2 mM ascorbic acid. Reduced phenolphthalein (rPHP) is very stable and was used as a LPMO substrate in the main text. Phenol red readily reduced to become transparent but also autoxidized within hours after pH neutralization. Fluorescein reduced reluctantly, similar to phenolphthalein (PHP), but showed weak autooxidation effect and sometimes precipitated during storage. Reduced fluorescein is a viable LPMO substrate, but it is not as stable as rPHP. Reduced crystal violet precipitated during zinc reduction, but it readily solubilized in strong acids or in 10% gelatine. When the gelatine gel was cast at pH 7.0 the reduced crystal violet autooxidised. Thymolphthalein blue reduced reluctantly, like PHP, but was not reoxidised by TaAA9A under the tested conditions. Bisphenol E did not make a simple coloured compound before or after reduction and was thus disregarded as a potential LPMO substrate.*

*Fig. S2: 2,7-Dichlorofluorescin diacetate (DCFH-DA) is a well-known cell-permeable compound that becomes fluorogenic inside cells that experience oxidative stress. Here we use DCFH-DA* in vitro *as a LPMO substrate at conditions similar to optimized assay conditions for rPHP oxidation. DCFH-DA, CAS 4091-99-0, was bought from Sigma-Aldrich and dissolved in DMSO to a concentration of 1 mM before further dilutions. For the assay, 10 µM DCFH-DA was incubated in 20 mM citrate-phosphate buffer pH 7.25 for 30 min and the production of the oxidation product, dichlorofluorescein, was followed continuously by fluorescence spectroscopy using excitation and emission wavelengths at 485 nm and 535 nm. The oxidation process was catalysed by 300 nM CuCl_2_ (green), 300 nM copper loaded TaAA9A (blue), and 300 nM copper loaded TaAA9A together with 100 µM DHA (red). The enhancing effect of adding DHA is evident, similarly to the observed effect with rPHP as the substrate.*


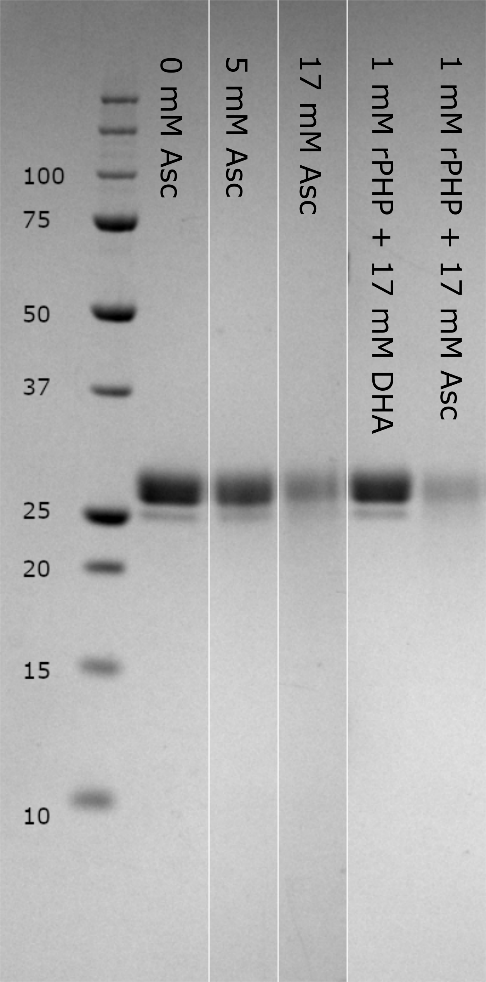

*Fig. S3: The effect of ascorbate in the assay: (****a****) Progress curves of ascorbate concentration in the rPHP assay was measured simultaneously with the progress curve of PHP production presented in Fig. 2a in the main text. Ascorbate has a strong UV band at 265 nm while its oxidized products does not and the change in A_265_ was used to qualify the amount of ascorbate in the rPHP assay conditions. rPHP was oxidised by 0.3 µM Cu-TaAA9A (orange), supplemented with 100 µM DHA (blue) or 100 µM ascorbate (green). Under these conditions, ascorbate is consumed within 10 min. There is a small increase in absorption with DHA, but this a result of the increase in PHP concentration. (****b****) Ascorbate induced degradation of TaAA9A as visualised on a SDS-PAGE gel. 10 µM Cu-TaAA9A was incubated in 25 mM citrate-phosphate buffer pH 7.25 for 30 min at 40 °C with various additions. A: MQ, B: 5 mM ascorbate, C: 17 mM ascorbate, D: 1 mM rPHP and 17 mM DHA, E: 1 mM rPHP and 17 mM ascorbate. Samples were mixed 4x XT sample buffer (Bio-Rad) containing 100 mM DTT, boiled at 100 °C for 5 minutes, and run at 144V for 1h.*

Gels where stained with Coomassie solution for 1h and de-stained overnight.

*Ascorbate at pH 7.25 clearly modifies TaAA9A in a dose dependent way that ultimately degrade it. Samples with DHA and rPHP were tested, but they essentially showed no difference in protein concentrations and only sample D with the combination of 1 mM rPHP and 17 mM DHA is shown. In contrast, the enzyme in sample E with the combination of 1 mM rPHP and 17 mM ascorbate has almost disappeared. Enzyme and substrate concentrations are higher than in the rPHP assay conditions and the two experiments are not directly comparable, but the ascorbate modification is evident and it is reasonable that the TaAA9A degradation is precluded by a faster modification that inactivate the enzyme but still makes it run as the unmodified enzyme on a SDS-PAGE gel. Omitted parts of the gel are indicated by white bars.*

*Fig. S4: Effects of buffer composition on the rPHP-based LPMO assay. 0.3 µM TaAA9A, 200 µM rPHP, and 100 µM DHA was incubated at 40* °*C for 30 min. Reactions were stopped by addition of 200 mM Na_2_CO_3_, pH 10.3 and the enzyme activity measured by absorption at 552 nm. (****a****) pH of several buffer systems (25 mM) were varied: Ethylmorpholine (red), MOPS (blue), citrate (black), phosphate (orange), citrate-phosphate (purple), citrate-phosphate-borate (green). (****b****) The rPHP assay with 25 mM citrate-phosphate-borate buffer series was repeated, but the absorption was measured without addition of Na_2_CO_3_. As expected, the assay gives a colour response at pH > 8 with increasing intensity as the pH goes past pKa = 9.4 of PHP. (****c****) Ionic strength in the rPHP assay was varied by the addition of 0-2 M NaCl to the 25 mM citrate-phosphate buffer, pH 7.25. The activity was stable to addition of 0-50 mM of NaCl. The effect on activity of adding higher concentrations of NaCl is shown in the insert.A NaCl inhibitory effectof IC50 ≈ 600 mM can be deduced. (****d****) Ionic strength in the rPHP assay was varied by changing the citrate-phosphate buffer concentrations 0-35 mM. The activity was fairly stable in the range of 5-35 mM. All data points were measured in triplicates and shown with standard deviations.*


Fig. S5 (**a**) *Effect of some co-substrates on the rPHP-based LPMO assay. (****a****) A basic rPHP assay: Triplicate samples of 0.3 µM TaAA9A, and 200 µM rPHP incubated at 40* °*C for 30 min. Reactions were stopped by addition of 200 mM Na_2_CO_3_ with pH = 10.3 and the enzyme activity measured by absorption at 552 nm. This condition was varied by addition of 100 µM DHA, ascorbate , BCA or EDTA as indicated under the graph bars.. In general, DHA enhances the assay output as described in the main text. Addition of the strong metal chelators BCA or EDTA does not affect the assay. (****b****) Uncoupled production of H_2_O_2_ by the rPHP assay was measured by co-incubation with the Amplex UltraRed assay-kit. Basic assay conditions: 200 µM rPHP and 100 µM DHA, incubated with the Amplex UltraRed reagent in 25 mM citrate-phosphate buffer pH 7.25 at 40 °C and measured as the fluorescent signal ex = 530 nm em = 590 nm. This condition was examined without a catalyst (blue), with 0.3 µM TaAA9A (green), or 0.3 CuCl_2_ (red). These conditions produce negligible amounts of H_2_O_2_. Rapid H_2_O_2_ production by 0.3 µM TaAA9A in similar conditions with 100 µM Asc is shown for comparison (purple). All data points were measured in triplicates and shown with standard deviations.*

*Fig. S6: PHP standard curve in assay conditions. 0-1 mM PHP samples were fourfold diluted in 25mM citrate-phosphate buffer pH 7.25 and the color developed by addition of Na_2_CO_3_. A_552_ absorption was measured similarly to how rPHP is handled in the assay (black circles). The absorbance at 552 nm was linearly proportional to the concentration of PHP with a slope 1.6 x 10^4^ M^-1^ (black line). The absorption spectrum scan of the 100 µM PHP sample is shown in the insert. Sample volumes are 250 µL in microtiter plates with a pathlength of 0.65 cm. In these assay conditions the extinction coefficient of PHP is ε_552_ = 2.5 x 10^4^ M^-1^. Red circles mark the absorption readings after complete oxidation of rPHP in the optimized rPHP assay conditions.*

*Fig. S7: HPAEC chromatograms of cellulose degradations products. 0.75 µM of Cu-TaAA9A was incubated with 0.5% PASC in citrate-phosphate buffers pH 3-8 at 50 ºC. (****a****) Reactions were supplemented with 1 mM ascorbate and stopped after 1 h by filtration. (****b****) Reactions were supplemented with 1 mM DHA and stopped after 1 h. (****c****) Reactions were supplemented with 1.25 mM fructose and stopped after 20 h. Ascorbate enabled PASC degradation at all pH values as expected. DHA enables PASC degradation, but preferably at pH > 5. Fructose enables PASC degradation at pH > 7, but does so much slower than ascorbate or DHA.*

*Fig. S8: Ascorbate oxidation rates are dependent on O_2_ concentration. The effect of oxygen concentration was measured on the BioTek Synergy H1 plate reader with a retro-fitted gas control unit. Oxidation of 100µM ascorbic acid catalyzed by 0.5 µM CuCl_2_(blue) and 0.5 µM Cu-TaAA9A (red). The reaction rates of the three oxygen conditions are not directly comparable due to a relatively long equilibration time of the assay which prohibited initial rate determinations.*
